# Validation of Aortic Blood Flow Simulations During Extracorporeal Circulation Using Phase Contrast Magnetic Resonance Imaging

**DOI:** 10.1111/aor.70136

**Published:** 2026-04-23

**Authors:** Anna Kathrin Assmann, Sinje Reimers, Jonah S. Schrauder, Lei Qu, Xiangge Yin, Amir Moussavi, Artur Lichtenberg, Susann Boretius, Michael Neidlin, Alexander Assmann

**Affiliations:** ^1^ Department of Cardiac Surgery Heinrich‐Heine‐University Düsseldorf Germany; ^2^ German Primate Center Functional Imaging Laboratory Göttingen Germany; ^3^ Department of Electrical Engineering and Information Technology South Westphalia University of Applied Sciences Hagen Germany; ^4^ Department of Cardiovascular Engineering, Institute of Applied Medical Engineering RWTH Aachen University Aachen Germany

**Keywords:** animal‐model, arterial wall, blood flow, computational fluid dynamics, extracorporeal circulation, phase contrast magnetic resonance imaging, wall shear stress

## Abstract

**Objectives:**

Although extracorporeal circulation (ECC) is routinely used in invasive cardiovascular medicine and can cause severe complications, the impact of ECC on arterial blood flow is not yet fully understood. This study aims to reveal actual bloodflow profiles during different ECC scenarios.

**Methods:**

Twenty‐three New Zealand White rabbits underwent ECC by ante‐ (*n* = 7) or retro‐grade (*n* = 9) or physiological perfusion (*n* = 7). Arterial blood flow profiles were assessed with a focus on cerebral and visceral perfusion. Numerical simulation models were tuned and validated based on magnetic resonance imaging (MRI).

**Results:**

Ante‐ and retrograde ECC resulted in completely divergent aortic blood flow patterns. Supraaortic and visceral perfusions were not impaired during ante‐ or retro grade ECC. Excellent correlation of volume flow rates was achieved between MRI and simulations (*r* = 0.98). Intima damage was observed in regions of high wall shear stress (WSS).

**Conclusions:**

This is the first study assessing arterial blood flow during different ECC scenarios in a living organism, and additionally validating precise blood flow simulations by in vivo measurements. Retrograde ECC does not a priori impair cerebral perfusion. Individualized simulations may guide cannulation strategies aiming at minimization of ECC‐related complications.

## Graphical abstract

This is the first study assessing in vivo blood flow profiles during extracorporeal circulation (ECC) scenarios. Magnetic resonance imaging data served for the calibration of computational fluid dynamics algorithms, enabling accurate replication of the hemodynamic conditions. Individualized simulations may guide cannulation strategies aiming at minimization of ECC‐related complications.

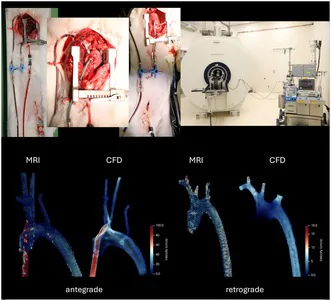

## 1 Introduction

Extracorporeal circulation (ECC) is routinely used in the majority of cardiothoracic operations, such as coronary bypass, heart valve or proximal aortic surgery, as well as for mechanical circulatory support (MCS) in patients with heart and/or lung failure, and the numbers are further increasing. Nevertheless, the ideal site of arterial cannulation (ascending aorta versus subclavian artery versus femoral artery, resulting in ante‐ or retrograde perfusion, respectively) is still under debate. There are scenarios in cardiac surgery which preclude femoral cannulation, such as status post aorto‐bi‐femoral prosthesis implantation, or thoracic cannulation, such as surgery via minimally invasive thoracotomies. However, for the majority of cardiac operations or MCS implantations, several strategies are possible so that the surgeon can choose the strategy with the presumably lowest risk of damage to the patient. Therefore, research on the impact of ECC on arterial blood flow and resulting complications is of utmost interest.

Due to so far missing in vivo models that exactly show bloodflow conditions during ECC, several computational models have been developed to simulate the blood flow in the human aorta and the hemodynamic impact of different cannula orientations on cerebral perfusion and arterial WSS [1, 2, 3, 4, 5, 6]. Computer simulations allow for the investigation of multiple different ECC flow conditions, thereby avoiding ethical and economic issues that would arise from high‐volume animal research. Moreover, computational models reveal flow patterns and resulting local WSS with higher precision as compared to current in vivo imaging modalities. To profit from the potential of computed simulations, validation of the simulation algorithms is required.

A considerably exact validation of a physiological blood flow simulation has been previously achieved based on 4D‐MRI data [7]. Quite challenging is the modulation of the boundary conditions of arterial vessels, which have a decisive impact on the simulation results. In this context, a regulatory model for physiological conditions in the human aorta has been implemented [8]. However, until now, mandatory validation of a computed model simulating ECC‐generated blood flow has not been performed.

In preparation of the current project, we have developed an MRI‐compatible small animal model of rabbits under ECC [9].

The primary aim of this study is to assess and comparatively examine arterial blood flow profiles during physiological blood flow versus thoracically applied antegrade ECC versus abdominally applied retrograde ECC by means of phase contrast magnetic resonance imaging (PC MRI). Afterwards, these data are used to validate blood flow simulation algorithms generated by computational fluid dynamics (CFD), and the effect of different cannulation strategies on aortic WSS and local vascular damage is assessed.

## 2 Material and Methods

### 2.1 Animals

Male New Zealand White rabbits (*n* = 26; 2500 g) were obtained from Charles River (Sulzfeld, Germany). All rabbits were fed ad libitum with standard chow. All experiments were conducted according to the “Guide for the Care and Use of Laboratory Animals,” and approved by the State Animal Care Committee (reference number: 33.19‐42502‐04‐21/3613).

### 2.2 Installation of ECC

**FIGURE 1:**
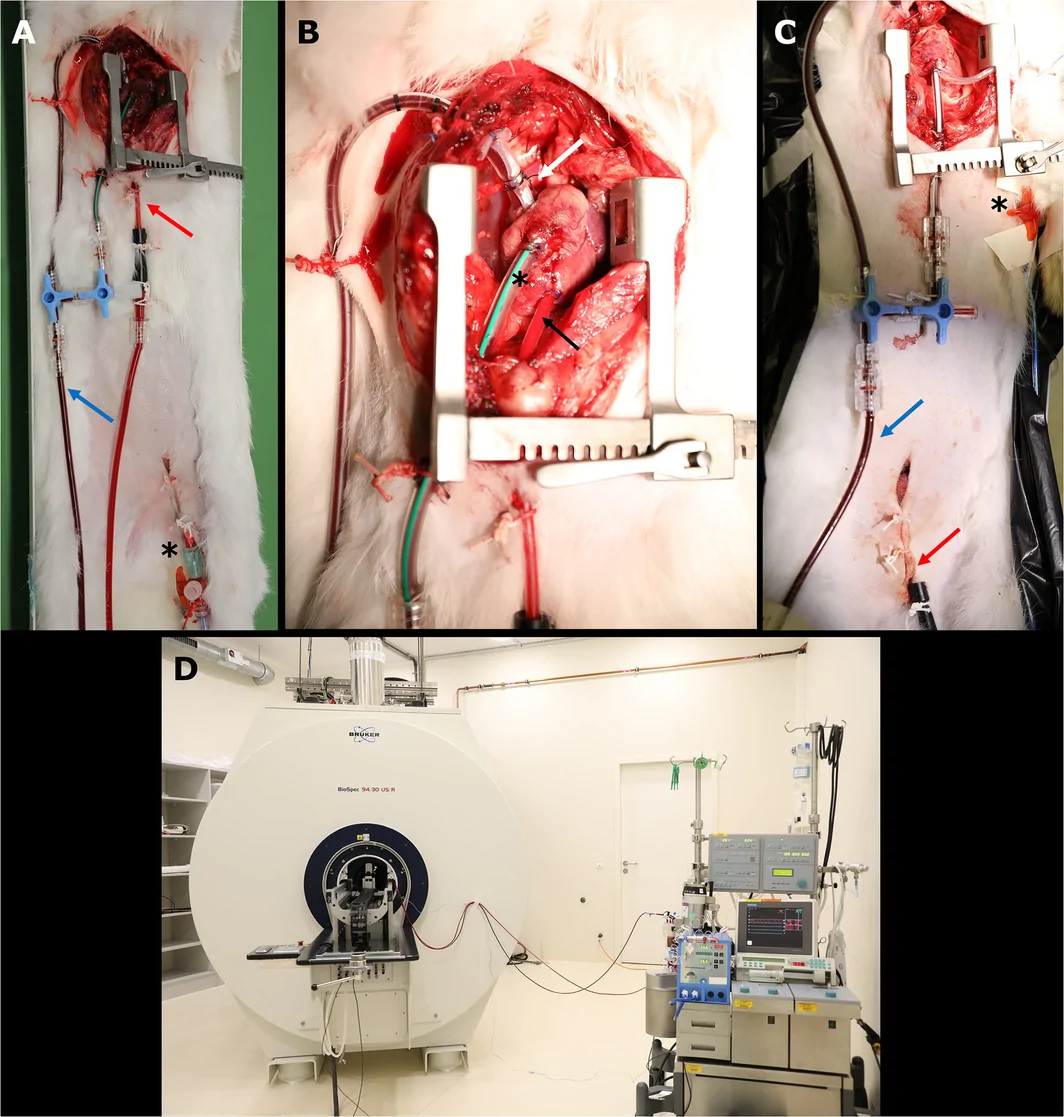
Experimental Setup of ECC installation and MRI transfer. (A) Operative setting for antegrade perfusion: arterial cannula (red arrow), venous drainage (blue arrow), cannula in the left femoral artery for invasive arterial pressure measurement (*). (B) Details of thoracic cannulation: venous cannula in the right atrium (white arrow), venous cannula in the right ventricle (*), arterial cannula (black arrow). (C) Operative setting for retrograde perfusion: cannula in the left brachial artery for invasive blood pressure measurement (*), venous drainage (blue arrow), arterial cannula (red arrow). (D) Setup during MRI measurement: magnetic resonance scanner (left side), heart‐lung machine (right side). [Color figure can be viewed at wileyonlinelibrary.com]

Anesthesia was induced with diazepam (2.5 mg/kg BW), ketamine (50 mg/kg BW) and xylazine (5 mg/kg BW), and maintained with 1%–1.5% isoflurane delivered through an endotracheal tube under mechanical ventilation. With the onset of ECC, isoflurane anesthesia was administered via the heart‐lung machine. For analgesia, rabbits got 0.02 mg/kg BW buprenorphine i.m. The left femoral artery (in case of physiological and antegrade perfusion) or the left brachial artery (in case of retrograde perfusion) was exposed, and a cannula was inserted for invasive blood pressure measurement (Figure 1A,C). Continuous electrocardiography, pulse oximetry and capnography were used to monitor vital parameters.

For the installation of ECC, a median sternotomy was performed. Systemic heparinization (100 IU/kg BW) was conducted targeting an activated clotting time > 400 s. For cannulation of the right atrium, a purse string stitch was conducted with a 5–0 Prolene suture, and after a small perforation, the venous cannula was inserted (Figure 1B). For better venous drainage, an additional venous line was inserted into the right ventricle (Figure 1B). For arterial cannulation, either the ascending aorta (for antegrade perfusion [Figure 1A,B]), or the abdominal aorta (for retrograde perfusion [Figure 1C]) was cannulated in the same manner as described above. The technique of cannulation and onset of the ECC is displayed in Video S1. In both cannulation scenarios, a Stöckert S2 heart‐lung machine (Stöckert, Freiburg, Germany), an infant oxygenator D100 Physio (Sorin, Milano, Italy), a hypothermia device (Stöckert, Freiburg, Germany), 2 × 8 m 1/8″ tubes, 14 Ch redon ‐drainages for venous drainage, and a 10 Ch catheter for arterial cannulation were used.

### 2.3 MRI‐Measurement and Analysis

Rabbits for analysis of physiological blood flow (*n* = 7), antegrade (*n* = 7) or retrograde (*n* = 9) ECC were transported into the MRI scanner to start the measurements (Figure 1D). Rabbits without any intervention or anesthesia (*n* = 3) served as controls for RNA analyses.

PC MRI flow data were acquired using a horizontal 9.4 T MR‐scanner (BioSpec, Bruker, Germany) with a 30‐cm bore size, equipped with a BGA‐20S gradient system and operated with Para Vision 6.0.1. The isotropic resolution was consistently maintained at 0.5 mm for all subjects, with a flip angle of 10°. In Data S1, detailed information about the methods of measurement and analysis can be found.

### 2.4 Blood Plasma Analysis

Blood was taken before and after ECC, or in case of physiological perfusion before and after MRI measurements. Serum levels of neuronspecific enolase (NSE) and S100B were analyzed at the Institute of Clinical Chemistry and Laboratory Diagnostics (Heinrich Heine University, Duesseldorf, Germany), applying commercially available standard assays designed for an automated clinical chemistry analyzer (series Cobas, Roche, Basel, Switzerland).

### 2.5 Explantation of Aorta and Brain

The aorta was explanted, and aortic rings were collected, rinsed with heparinized PBS and processed for (immuno‐)histology. Additionally, the brain of the rabbits was explanted for PCR and stored at −80°C.

### 2.6 Immunohistology

Data S2.

### 2.7 Quantitative RNA‐Analysis

Data S3.

### 2.8 Simulations

Aortic blood flow was determined through CFD with the ANSYS‐Workbench 2024R1 software (Ansys Inc., Gettysburg, PA, USA). A detailed description can be found in Data S4 and S5.

In total, *n* = 4 aortic arch geometries with antegrade perfusion and *n* = 4 aortic arch geometries with retrograde perfusion were analyzed. In addition, flow in the abdominal aorta (AoAbd) was analyzed for *n* = 6 animals (3 antegrade and 3 retrograde) as two datasets had to be excluded due to low quality of the segmented vessels. Physiological flow for the WSS evaluation was performed for *n* = 8 animals.

### 2.9 Statistics

Variables are shown as median and (interquartile range [IQR]). Direct group comparisons were conducted by Mann–Whitney *U*‐tests. Multiple group comparisons were conducted by Kruskal–Wallis tests with Dunn post hoc tests. Comparisons between CFD and PC MRI were performed using Pearson correlation and Bland–Altman analysis to assess agreement and systematic bias. A *p* < 0.05 was considered statistically significant. Data analysis was conducted with GraphPad Prism8 (GraphPad Software, San Diego, CA, USA).

## 3 Results

### 3.1 Hemodynamics and Oxygenation Under ECC

Anesthesia and MR measurements were successful without complications in all rabbits with physiological perfusion only (*n* = 7). Installation of ECC was successful without complications in all animals with either antegrade (*n* = 7 [Figure 1A,B]) or retrograde (*n* = 9 [Figure 1C]) perfusion. Hemoglobin levels significantly dropped after the onset of ECC (*p* < 0.0001) reaching constant levels during the MRI measurements (Figure S1A). Also, hematocrit levels significantly dropped after the onset of ECC (*p* < 0.0001) reaching constant levels during the MRI measurements (Figure S1B). Blood flow rates generated by the heart‐lung machine were constant during the whole experiment (Figure S1C). Mean arterial pressure (MAP) was constant during ECC (Figure S1D). PaO_2_ was adequate before and during ECC (Figure S1E), as well as SpO_2_ (Figure S1F).

### 3.2 Distribution of Arterial Blood Flow

**FIGURE 2:**
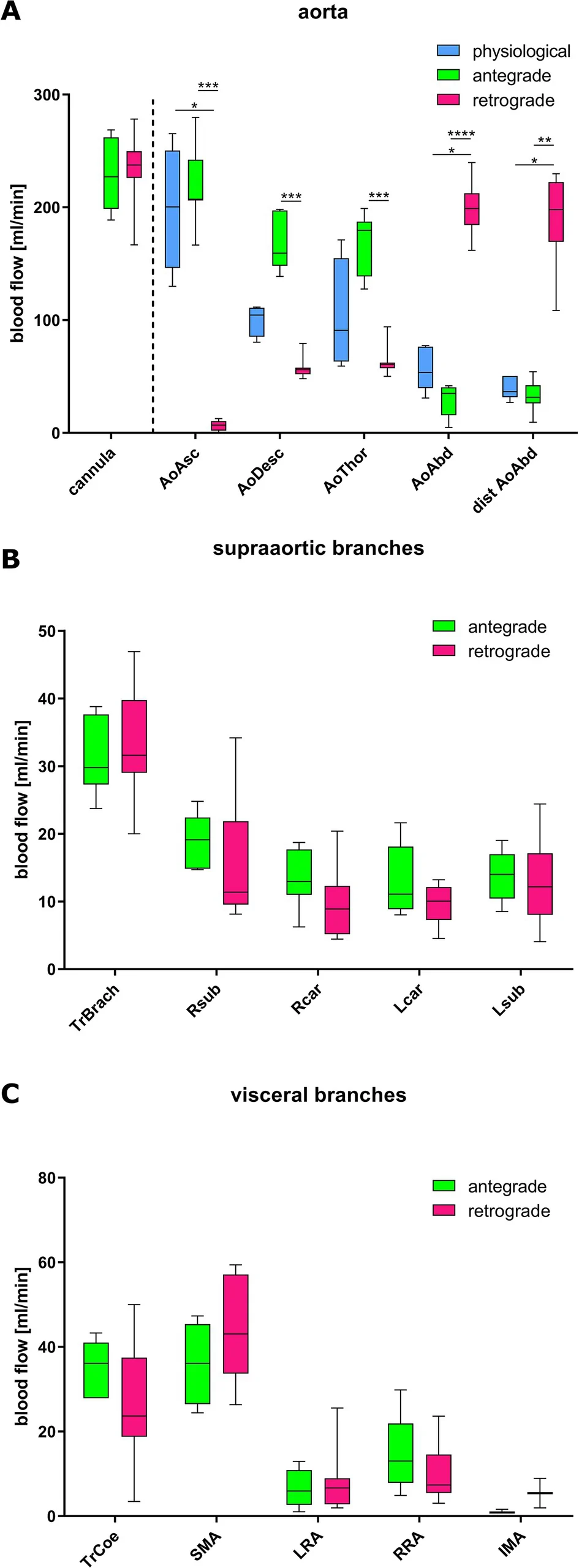
Blood flow distribution during physiological and extracorporeal circulation. (A) Comparison of blood flow distribution for physiological, antegrade and retrograde perfusion. (B) Comparison of blood flow distribution to the supraaortic branches during antegrade and retrograde perfusion. (C) Comparison of blood flow distribution to the visceral branches during antegrade and retrograde perfusion. *p < 0.05, **p < 0.01, ***p < 0.001, ****p < 0.0001. [Color figure can be viewed at wileyonlinelibrary.com]

During antegrade and retrograde perfusion, the inflow volume through the arterial cannula was similar in both groups during the whole experiments (*p* = 0.76 [Figure 2A]). Blood flow in the ascending aorta (AoAsc) was comparable between physiological and antegrade perfusion (*p* > 0.9999 [Figure 2A]), whereas the blood flow of retrograde perfusion was significantly lower compared to physiological (*p* = 0.05 [Figure 2A]) and antegrade perfusion (*p* = 0.0008 [Figure 2A]). Compared to physiological perfusion, blood flow in the descending aorta (AoDesc) and thoracic aorta (AoThor) was also comparable during antegrade (AoDesc: *p* = 0.41; AoThor: *p* = 0.35 [Figure 2A]) and not significantly lower during retrograde perfusion (AoDesc: *p* = 0.2; AoThor: *p* = 0.46 [Figure 2A]). Blood flow volume in the AoAbd and distAoAbd was comparable between physiological and antegrade perfusion (AoAbd: *p* = 0.78; distAoAbd: *p* > 0.9999 [Figure 2A]), whereas the blood flow during retrograde perfusion was significantly higher compared to physiological flow (AoAbd: *p* = 0.05; distAoAbd: *p* = 0.017 [Figure 2A]).

Neither for the perfusion in the supraaortic branches (brachiocephalic trunc [TrBrach], right subclavian artery [Rsub], right carotid artery [Rcar], left carotid artery [Lcar] and left subclavian artery [Lsub]); nor for the visceral branches (coeliac trunk [TrCoe], superior mesenteric artery [SMA], left renal artery [LRA], right renal artery [RRA], inferior mesenteric artery [IMA]) differences occurred comparing antegrade with retrograde perfusion (Figure 2B,C).

**FIGURE 3:**
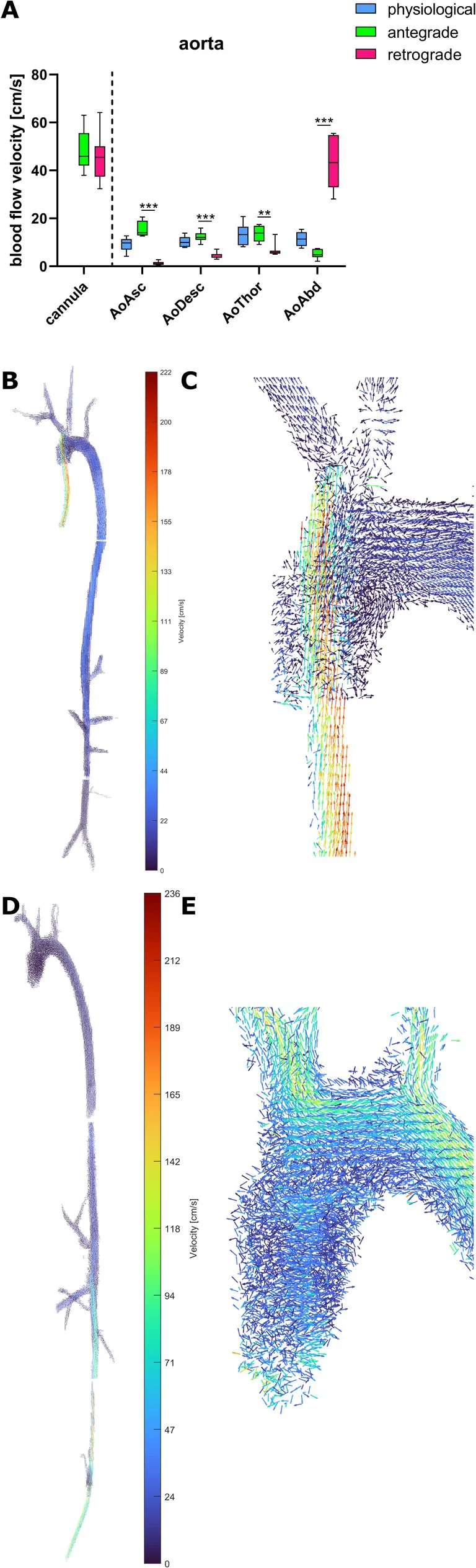
Blood flow velocity during physiological and extracorporeal circulation. (A) Comparison of blood flow velocity for physiological, antegrade and retrograde perfusion. (B) Representative pattern of blood flow velocity in the aorta during antegrade perfusion. (C) Detailed velocity vector graphic displaying the cannula jet blood stream including turbulences in the AoAsc and proximal aortic arch during antegrade perfusion. (D) Representative pattern of blood flow velocity in the aorta during retrograde perfusion. (E) Detailed velocity vector graphic of blood stream in the AoAsc and proximal aortic arch during retrograde perfusion. **p < 0.01, ***p < 0.001. [Color figure can be viewed at wileyonlinelibrary.com]

The blood flow velocity of the inflow cannulas during antegrade and retrograde perfusion was similar (*p* = 0.76 [Figure 3A]). In the AoAsc, the blood flow velocity was significantly higher during antegrade compared to retrograde perfusion (*p* = 0.0002 [Figure 3A]). In the AoDesc, significantly lower blood flow velocity occurred during retrograde compared to antegrade perfusion (*p* = 0.0003 [Figure 3A]), which was also detected in the distAoThor (Figure 3A). In the AoAbd, compared to antegrade perfusion a significantly higher blood flow velocity was found during retrograde perfusion (*p* = 0.0002 [Figure 3A]).

A qualitative comparison of blood flow velocity vectors during antegrade and retrograde perfusion is shown in Figure 3B–E. Especially in the AoAsc and aortic arch, turbulences occur due to the jet stream of the inflow cannula during antegrade, which is in opposite to retrograde perfusion (Figure 3C).

### 3.3 Inflammation and Brain Injury Biomarkers

In the blood plasma analyses, NSE‐ and S100‐levels were similar in all groups before and during ECC, or in case of physiological perfusion before and after MRI measurements (Figure S2A,B).

Inflammatory activity in the brain was assessed by PCR. No differences were detected for IL‐6 and TNF‐alpha gene expression between controls and the three intervention groups (Figure S2C,D). Also, for the brain injury‐associated gene markers IBA1, NSE and S100B no differences were found (Figure S2E–G).

### 3.4 Aortic Intima Damage

**FIGURE 4:**
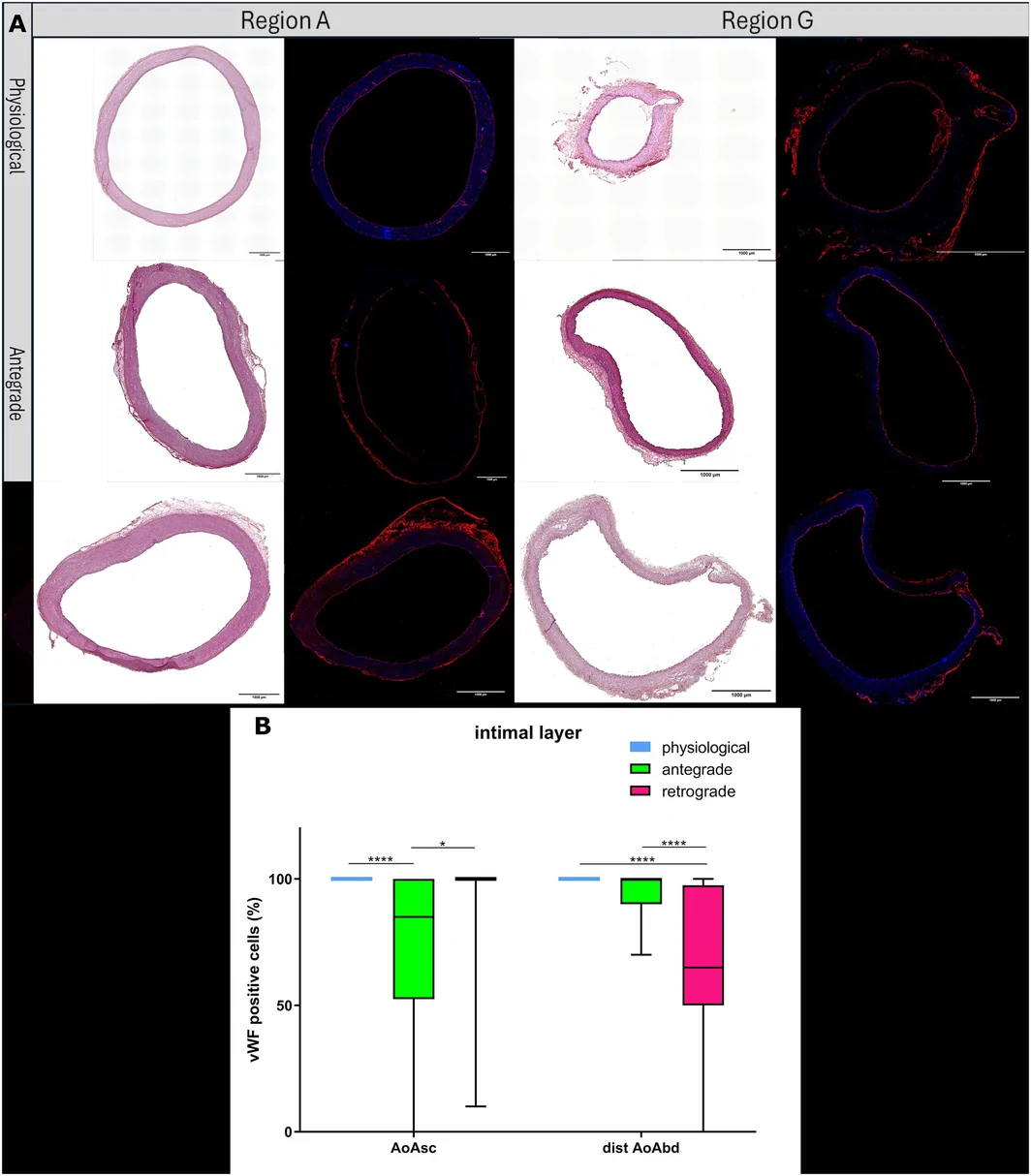
Analysis of the aortic intimal layer. (A) Representative hematoxylin–eosin staining with corresponding vWF‐staining of the AoAsc (region A) and distAoAbd (region G) of rabbits with physiological, antegrade and retrograde perfusion. (B) Comparison of vWF positive cells in the AoAsc and distAoAbd of rabbits with physiological, antegrade or retrograde perfusion.*p < 0.05, ****p < 0.0001. [Color figure can be viewed at wileyonlinelibrary.com]

Intimal layers of AoAsc and AoAbd, which were close to the inflow cannulas, were analyzed [Figure 4A]. The aortae of rabbits without heart‐lung machine showed an intact intima with 100% vonWillebrand factor (vWF) positive cells. Significantly less vWF positive cells were detected in the AoAsc in rabbits with antegrade compared to rabbits with physiological (*p* < 0.0001 [Figure 4B]) and retrograde perfusion (*p* = 0.01 [Figure 4B]). In the distAoAbd, significantly less vWF positive cells were seen in rabbits with retrograde compared to rabbits with physiological (*p* < 0.0001 [Figure 4B]) or antegrade perfusion (*p* < 0.0001 [Figure 4B]).

### 3.5 MRI‐Based Validation of CFD‐Simulations

**FIGURE 5:**
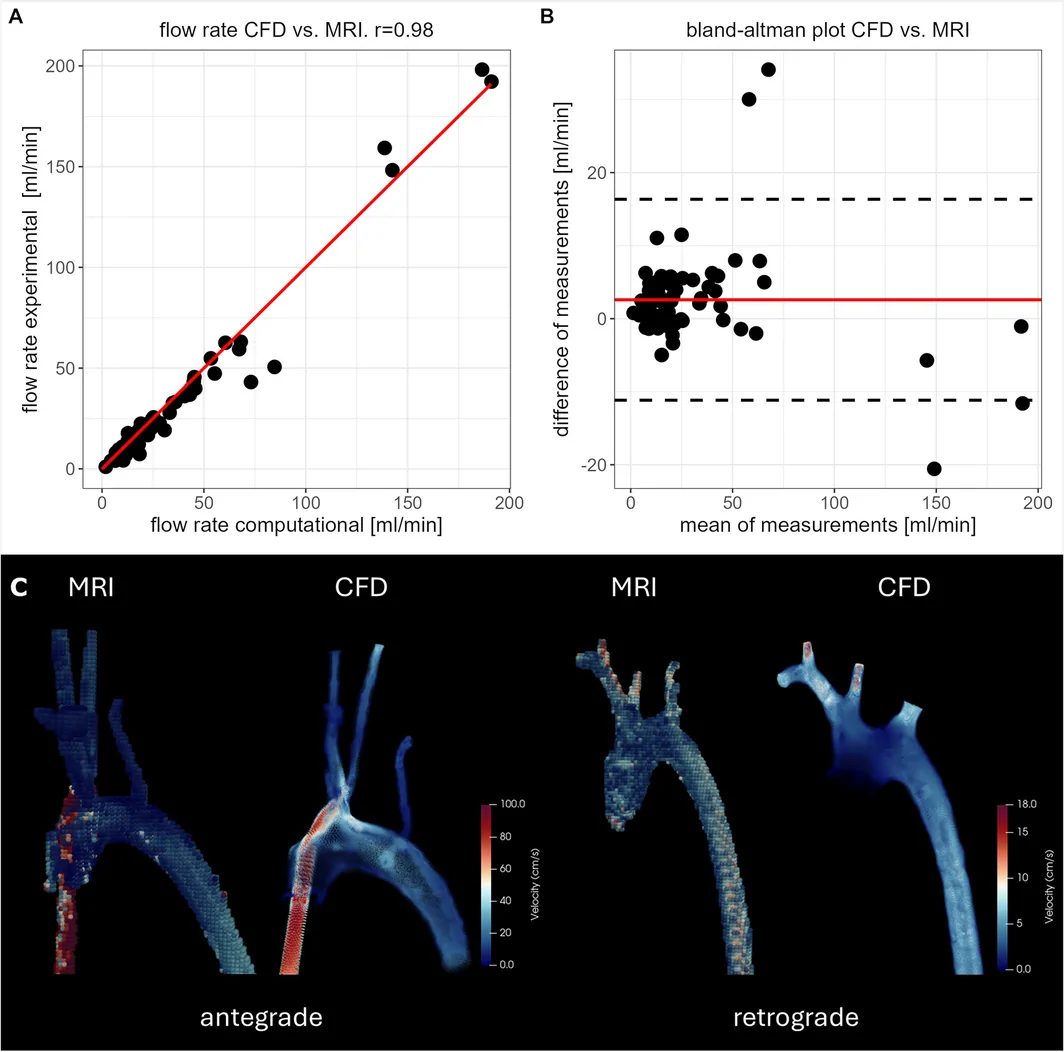
Comparison of blood flow rates and velocities between CFD and MRI measurements. Correlation plot (A) and Bland–Altman plot (B) for the comparison of blood flow rates at the model outlets between CFD and PC MRI measurements. Qualitative comparison of velocity fields obtained from PC MRI and CFD for representative antegrade and retrograde cannulation scenarios (C). [Color figure can be viewed at wileyonlinelibrary.com]

Figure 5 shows the comparison of the volume flow rates between PC MRI and CFD at the outlets of the aortic model in a correlation plot in Figure 5A and a Bland–Altman plot in Figure 5B. An excellent correlation (*r* = 0.98) was observed, and the Bland–Altman plot shows a small bias (2.6 mL/min) and only 3 out of 66 measurements outside the limits of agreement.

A qualitative comparison of the velocity magnitude is shown in Figure 5C. The spatially resolved velocities correspond well between PC MRI and CFD with similar peak values and distributions. CFD allows for substantially higher spatial resolution, resulting in more detailed flow information particularly in regions with complex flow patterns such as the area where the jet hits the aortic wall.

**FIGURE 6:**
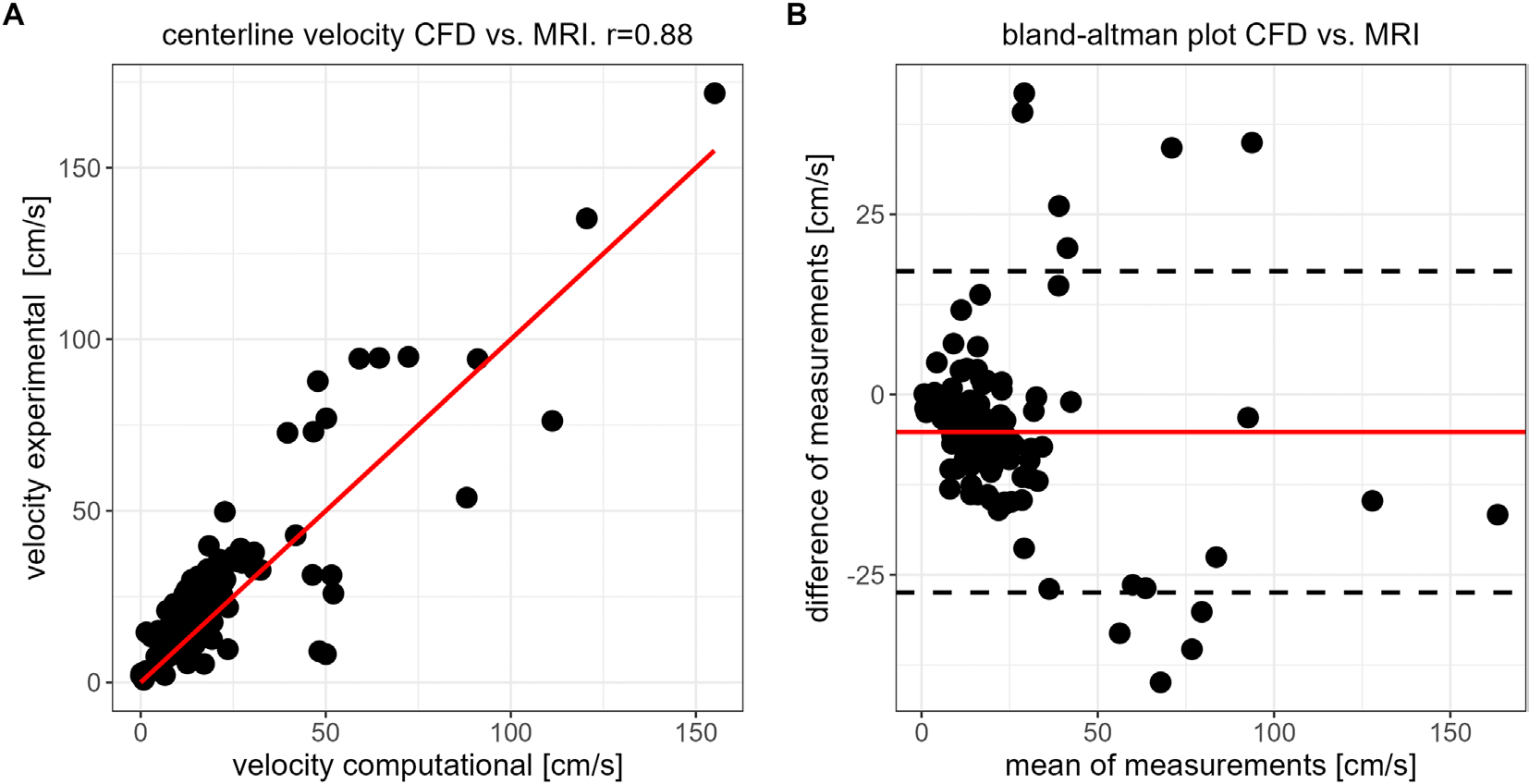
Comparison of aortic centerline velocities between CFD and MRI. Correlation plot (A) and Bland–Altman plot (B) for the comparison of centerline velocities for the aortic arch and AoAbd between CFD and PC MRI measurements. [Color figure can be viewed at wileyonlinelibrary.com]

The comparison of the aortic centerline velocities is shown for all animals and anatomies (aortic arch = 8, AoAbd = 6) in Figure 6. Figure 6A displays the very good correlation of *r* = 0.88 between CFD and PC MRI data. The Bland–Altman plot in Figure 6B presents a small bias of −5 cm/s and 10 out of 145 data points outside the limits of agreement (~7%).

### 3.6 Wall Shear Stress

**FIGURE 7:**
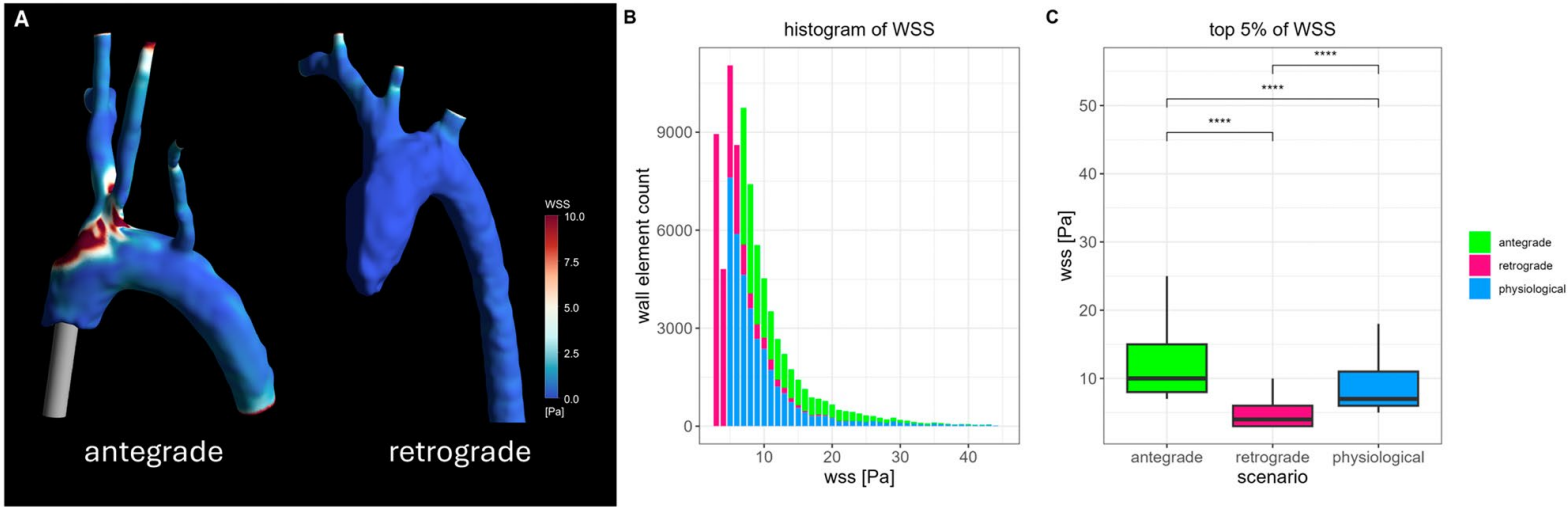
Wall shear stress. (A) Comparison of WSS patterns for representative antegrade and retrograde cannulation scenarios. (B) Histogram of WSS values for all animals. (C) Boxplots of the top 5% of WSS values for antegrade, retrograde perfusion and physiological flow at peak‐systole. ****p < 0.0001. [Color figure can be viewed at wileyonlinelibrary.com]

CFD‐based WSS comparisons for the different cannulation scenarios are shown in Figure 7. Figure 7A displays the WSS distribution pattern during antegrade and retrograde perfusion for the same animals as in Figure 5C. Antegrade perfusion shows higher WSS values than retrograde perfusion with the peak values at the location where the cannula‐jet hits the wall. Figure 7B shows the number of wall‐mesh‐elements with WSS from 0 to 40 Pa for all scenarios. Antegrade perfusion exhibits a rightward shift of the WSS values, while retrograde perfusion has the largest amount of elements with low WSS values. To statistically analyze the differences, we extracted the top 5% WSS per category (*n* = 4 antegrade, *n* = 4 retrograde, *n* = 8 physiological) and compared all groups (Figure 7C). Antegrade perfusion exhibits the highest WSS with a median value of 10 Pa that is significantly higher than for retrograde (median = 4 Pa) or physiological flow (median = 7 Pa), with *p* < 0.0001 for all comparisons. In addition, it was observed that retrograde perfusion yields significantly lower WSS values than physiological peak systolic perfusion.

## 4 Discussion

Heart‐lung machines are life‐saving medical devices for a multitude of cardiothoracic surgeries, interventions and MCS therapies, such as extracorporeal life support (ECLS) and extracorporeal membrane oxygenation (ECMO). Understanding changes in blood flow dynamics due to ECC is essential to improve patients' outcomes, since ECC by heart‐lung machines exhibits an enhanced risk of severe complications, such as vascular injury or stroke. In this context, several studies have used CFD which certainly have the potential to display detailed blood flow conditions during ECC scenarios; however, the simulation algorithms and resulting flow patterns have not been validated by actual measurements yet [1, 3, 4, 5, 10]. The underlying reason is that until now, no in vivo data of blood flow profiles during ECC have existed.

In the present study, our MRI‐compatible small animal model [9] was tuned to examine aortic blood flow profiles in rabbits during different ECC conditions and additionally compare these data to physiological blood flow. Afterwards, MRI data were used to validate blood flow simulations, which additionally allowed for the calculation of WSS patterns.

During the whole ECC experiment, all rabbits showed adequate systemic perfusion with constant MAP, SpO_2_ and paO_2_, which underlines the feasibility and reproducibility of this complex animal model in terms of adequate hemodynamics and oxygenation.

As expected, blood flow during antegrade perfusion was similar to physiological perfusion except for missing pulsatility during ECC whereas blood flow during retrograde perfusion was significantly lower in the AoAsc and higher in the AoAbd. Despite these logical findings, the blood flow distribution to the supraaortic and to the visceral branches showed no differences between ante‐ and retrograde perfusion. This fact is of utmost interest, as until now it was not known if the organ perfusion during retrograde perfusion is as effective and sufficient as during antegrade or physiological perfusion. Especially the brain perfusion during ECC is a quite controversially discussed topic. Certainly, neurological impairment due to hypoperfusion, hypoxemia, intracranial hemorrhage, thrombo‐emboli, edema or impairment of cerebral autoregulation is one of the most feared complications during ECC [11]. During antegrade perfusion, the blood flow is similar to physiological perfusion, so it is likely that the brain has a sufficient perfusion, whereas during retrograde perfusion, the blood flows in reverse direction in the whole abdominal and thoracic aorta before it reaches the supraaortic branches. Our results demonstrate that nevertheless, the blood flow in the supraaortic branches is not different compared to antegrade perfusion, leading to the conclusion that cerebral perfusion is not a priori impaired by retrograde ECC. This result is extremely valuable in the context of minimally invasive surgery that frequently technically requires ECC by retrograde perfusion.

A relevant readout aspect addressing tissue damage by potential malperfusion is the inflammatory activity in the tissue. Here, we show that neither the inflammatory activity in the brain nor typical brain‐injury biomarkers in the blood were enhanced neither in rabbits with a long anesthesia and physiological perfusion nor in rabbits with ECC compared to control rabbits without any intervention or anesthesia. This outcome underlines again the hemodynamic stability in the animal model as well as the hypothesis that antegrade and retrograde perfusion both are safe and effective perfusion strategies. So far, neuromonitoring during ECC is the only tool to intraoperatively detect potential malperfusion of the brain, and brain‐injury‐associated biomarkers in the blood support the diagnosis postoperatively‐however, even these options are not used in all heart centers [12]. Reports from an international survey on neuromonitoring and neurodevelopmental outcome in children and adults supported by ECMO in Europe yielded, that out of 133 European ECMO centers only 43% use routine neuromonitoring during ECMO, the majority of which were near‐infrared spectroscopy (NIRS) (66.2%), electroencephalography (39.1%), transcranial Doppler in children (28.5%) or brain‐injury biomarkers in the blood (24.8%) [13]. Neuromonitoring during ECC has to be further improved ‐ qualitatively as well as quantitatively.

Local vascular lesions are typical complications of ECC with partially fatal outcomes, such as arterial dissection, local thrombus generation with subsequent embolization, or plaque embolization in case of aortic atherosclerosis [14, 15, 16, 17]. In this context, high blood velocity that stresses the aortic wall is an important source of wall damage. In our experiments, during antegrade perfusion, the velocity pattern was similar to physiological perfusion. During retrograde perfusion, the blood velocity in the thoracic aorta was significantly lower as compared to antegrade perfusion, whereas the opposite was true in the abdominal aorta. Compatible with these observations, we have found an intact intimal layer with ubiquitously vWF‐positive cells in the aorta without ECC, whereas during antegrade perfusion, the intimal layer in the ascending aorta showed significantly less vWF‐positive cells, hinting at the mechanism that the jet stream from the ECC cannula may have injured the intimal layer in this region. In line with these findings, we detected fewer vWF‐positive cells in the distal abdominal aorta during retrograde perfusion as compared to physiological and antegrade perfusion.

The present study also features the validation of CFD algorithms that have the power to precisely simulate ECC scenarios. The comparison of volume flow rates between PC MRI measurements and CFD simulations shows excellent correlation at multiple defined levels along the aorta and its branches. Furthermore, we demonstrate that the velocities correspond well between MRI and CFD with similar peak‐values and flow vector patterns. Also, the centerline velocities in the aorta are equivalent in MRI and CFD. Overall, the here developed CFD algorithms adequately simulate the blood flow conditions in rabbits during ECC. However, the current CFD model uses parameters for the boundary conditions (loss coefficients) that have been iteratively adopted to the flow rate measurements from PC MRI. These coefficients depend on the anatomy and the downstream vasculature of the animals and exhibit large variations between the individual animals. Using cohort averaged values for the loss coefficients did not yield convincing results. From a translational point of view, the arterial boundary conditions of a patient would have to be determined based on the individual vascular anatomy. In fact, identification of patient‐specific coefficients from clinical data is a well‐known problem [18]. In practice, these coefficients would be determined from non‐invasive flow rate measurements using PC MRI or Doppler echocardiography before ECC is applied. Subsequently, different cannulation scenarios can be compared on a patient‐specific basis.

PC MRI blood flow measurements provide diverse information on blood flow patterns and flow distribution during physiological and ECC perfusion. Nonetheless, PC MRI measurements have limitations which justify the use of computer simulations. First of all, until now, MRI measurement of ECC‐perfusion in humans is not possible due to the fact that heart‐lung machines have metallic components and thus do not enable MRI measurements. Furthermore, in contrast to MRI, validated CFD algorithms have the potential to precisely simulate ECC scenarios within a relatively short period of time, thereby avoiding complex experimental setups and ethical issues in the context of research on animals or human beings. Additionally, MRI measurements cannot provide exact information on WSS—in contrast to simulations. This becomes even more challenging in case of turbulent flows with high velocities and complex three‐dimensional flow‐structures, all of which are expected to be present in ECC perfusion. Although numerical simulations need careful experimental validation, their utilization as a predictive tool for cardiovascular medicine has great potential [19]. Previous studies have shown that increased WSS may cause rupture of atherosclerotic plaques and thus lead to embolic complications [4, 20, 21, 22, 23]. In the present study, WSS was significantly increased during antegrade perfusion in the ascending aorta compared to retrograde perfusion and even increased as compared to peak‐systole values of physiological blood flow. Antegrade perfusion overall exhibited twice as high WSS values. Especially in living organisms with atherosclerotic plaques this information is of utmost importance as atherosclerosis is a major risk factor for stroke [24]. One of the main mechanisms that cause embolism in humans is the cannula blood flow jet hitting the aortic wall [25, 26, 27], so that the positioning of the outflow cannula in relation to existing atherosclerotic plaques in the aorta may be decisive. CFD simulations can individually guide the cannula positioning to avoid the above‐mentioned complications.

## 5 Conclusion

In conclusion, the present study establishes a reproducible MRI compatible small animal model that enables, for the first time, in vivo assessment of aortic blood flow profiles during ECC. The results demonstrate that both antegrade and retrograde perfusion strategies provide stable systemic and cerebral perfusion, with comparable flow distribution to supraaortic and visceral branches. These findings suggest that retrograde perfusion, despite its reversed flow direction, does not inherently compromise organ or brain perfusion under the investigated conditions, supporting its clinical applicability in minimally invasive cardiothoracic procedures.

Furthermore, the study provides the first experimental validation of CFD simulations by in vivo MRI flow measurements during ECC. The strong agreement between PC MRI data and CFD simulations confirms that sophisticated computational models can reliably reproduce complex hemodynamic conditions during extracorporeal support. Importantly, the simulations enabled detailed assessment of wall shear stress patterns, revealing elevated WSS during antegrade perfusion in the ascending aorta, which may contribute to vascular injury or embolic complications, particularly in the presence of atherosclerotic disease.

Together, these findings highlight the translational potential of combining MRI‐based flow measurements with validated CFD simulations to improve the understanding of hemodynamics during ECC. In the future, patient‐specific computational modeling may support individualized cannulation strategies and optimize perfusion settings, thereby reducing complications and improving outcomes in patients requiring MCS.

## Author Contributions

A.K.A.: writing – original draft, conceptualization, funding acquisition, investigation, methodology, validation, visualization. S.R.: data curation. J.S.S.: data curation, visualization. L.Q.: data curation. X.Y.: data curation. A.M.: writing – review and editing. A.L.: writing – review and editing. S.B.: writing – review and editing. M.N.: data curation, visualization, writing – review and editing. A.A.: conceptualization, formal analysis, supervision, project administration, funding acquisition, validation, methodology, investigation, writing – review and editing.

## Funding

Supported by German Research Foundation (AS702/1‐1) and German Heart Foundation/German Foundation of Heart Research (F/18/20).

## Conflicts of Interest

The authors declare no conflicts of interest.

## Supporting information


**Data S1:** aor70136‐sup‐0001‐Supinfo.docx.

**Figure S1:** Hemodynamic parameters and oxygenation under ECC. (A) Hemoglobin levels (median + IQR) before and during extracorporeal circulation (ECC). (B) Hematocrit levels (median + IQR) before and during ECC. (C) Blood flow rates (median + IQR) during ECC. (D) Mean arterial pressure (MAP) (median + IQR) before and during ECC. (E) Arterial partial pressure of oxygen (paO_2_) (median + IQR) before and during ECC. (F) Peripheral oxygen saturation (SpO_2_) (median + IQR) before and during ECC.
**Figure S2:** Inflammatory activity and brain damage markers. (A) Comparison of neuronspecific enolase (NSE) in rabbits with physiological blood flow, antegrade or retrograde perfusion before and after the MRI measurement. (B) Comparison of S100 calcium‐binding protein (S100) in rabbits with physiological blood flow, antegrade or retrograde perfusion before and after the MRI measurement. (C) Interleukin‐6 gene expression of controls, of rabbits with physiological blood flow, antegrade or retrograde perfusion. (D) Tumor necrosis factor alpha (TNF alpha) gene expression of controls, of rabbits with physiological blood flow, antegrade or retrograde perfusion. (E) Ionized calcium‐binding adapter molecule 1 (IBA1) gene expression of controls, of rabbits with physiological blood flow, antegrade or retrograde perfusion. (F) NSE gene expression of controls, of rabbits with physiological blood flow, antegrade or retrograde perfusion. (G) S100b gene expression of controls, of rabbits with physiological blood flow, antegrade or retrograde perfusion.

**Table S1:** Primer sequences for quantitative RT‐PCR.

**Video S1:** Experimental‐setup of ECC‐installation + MRI‐transfer.

## Data Availability

The data that support the findings of this study are available on request from the corresponding author. The data are not publicly available due to privacy or ethical restrictions.
