# Supplementary material for: Validation of Aortic Blood Flow Simulations During Extracorporeal Circulation Using Phase Contrast Magnetic Resonance Imaging
Source: Artif Organs. 2026 Apr 23;50(9):1217–27. doi: 10.1111/aor.70136 (PMC13525229; doi:10.1111/aor.70136)
Supplement: Supplementary file 1 [file AOR-50-1217-s001.docx]

**Supplementary Data 1**

For subjects connected to ECC, three separate velocity-encoded (Venc) acquisitions were conducted at flow velocities of 200, 50, and 20 cm/s to capture a broad dynamic range. Data from each measurement were obtained separately and subsequently combined during post-processing. To minimize the scan-time, the echo time (TE) and repetition time (TR) were reduced to a minimum of 3.4 ms and 7.0 ms, respectively. This resulted in a total scan-time of approximately 12minutes for each acquisition, depending on the size of the animal and the region of interest. The acquisition bandwidth was set to 200 kHz.

For subjects not connected to ECC, a modified version of Bruker's FLOWMAP sequence was employed to capture physiological cardiac flow. This sequence was adapted by incorporating a navigator before the flow-encoding-gradients, which allowed for the retrospective gating of the data into pseudo-dynamic cardiac phases during post-processing. Due to the requirement for the navigator, the TE and TR were extended to 4.0 ms and 11.0 ms, respectively, and the acquisition bandwidth was adjusted to 100 kHz.

**Supplementary Data 2**

For histology, aortic cryosections (5 µm thick) were used to perform hematoxylin–eosin staining for morphometrical analyses.

For immunohistology, aortic cryosections (5 µm thick) were incubated at room temperature for 10 minutes with 0.25% Triton X-100 and for 1 hour with 5% bovine serum albumin + 0.1% Tween-20. Incubation with a primary antibody detecting vonWillebrand Factor (vWF) (Abcam, Cambridge, UK) + 1% bovine serum albumin + 0.1% Tween-20 lasted 1 hour at 37°C. A secondary antibody conjugated to the fluorophore Alexa 546 (Invitrogen, Carlsbad, CA, USA) was applied (in PBS containing 1% bovine serum albumin and 0.1% Tween-20) for 45 minutes in a dark and humid chamber at 37°C. For counterstaining, cryosections were incubated with 4´,6-diamidino-2-phenylindole (DAPI) (Roth, Karlsruhe, Germany) for 5 minutes in a dark and humid chamber. Afterwards, sections were covered with Leica medium (Leica Biosystems, Nussloch, Germany), and image acquisition was conducted with a microscope system DM2000, equipped with a digital camera DFC 425C (Leica, Wetzlar, Germany) and the Leica Application Suite V3.7 software. Analyses for the detection of intergroup differences were performed using Image J (Wayne Rasband, National Institutes of Health, Bethesda, MD, USA).

**Supplementary Data 3**

Quantitative real time (RT)-PCR was performed to analyze marker gene expressions in the explanted brain. In addition to the three intervention groups (physiological perfusion (n=3), antegrade perfusion (n=3) and retrograde perfusion (n=3)), rabbits without any procedure or anesthesia were taken as reference group (n=3).

Total RNA was isolated using a commercially available kit (RNeasy Mini Kit, Qiagen, Hilden, Germany). In brief, tissue was homogenized in TRIzol (Sigma-Aldrich, Steinheim, Germany), and RNA was precipitated by isopropanol. To conduct quality analyses, RNA samples were collected after passaging through a DNA-removing column (Qiagen, Hilden, Germany). Quantity as well as purity of the isolated RNA were determined spectrophotometrically (BioPhotometer plus; Eppendorf, Hamburg, Germany), and optical density values at 230, 260, and 280 nm were recorded. Furthermore, to detect the level of RNA degradation, the Agilent RNA 6000 Nano Kit (Agilent Technologies, Santa Clara, CA, USA) was used. Total cDNA was generated using QuantiTect Reverse Transcription Kit (Qiagen, Hilden, Germany) according to the manufacturer’s recommendation. Quantitative RT-PCR was conducted on a StepOnePlus cycler (Applied Biosystems, Foster City, CA, USA) using the Platinum SYBR Green PCR Master Mix (Invitrogen, Darmstadt, Germany) with a reaction volume of 20 µl: 50°C for 2 minutes, 95°C for 2 minutes, 95°C for 15 seconds and 60°C for 30 seconds (40 cycles), 95°C for 15 seconds, 60°C for 1 minute, 95°C for 15 seconds and 60°C for 15 seconds.

Relative gene expression was determined using the ΔΔCt method and beta-2 microglobulin (β2M) as housekeeping gene. Primers for the following genes were obtained from Invitrogen: inflammation-associated gene markers [tumor necrosis factor alpha (TNF-alpha) and interleukin 6 (IL-6)] and brain injury-associated gene markers [ionized calcium-binding adapter molecule 1 (IBA1), neuronspecific enolase (NSE) and S100 calcium-binding protein B (S100B)]. In [Supplementary Table 1], all primer sequences are presented.

**Supplementary Data 4**

At first, the segmented STL files of the rabbit aortae were processed and smoothed before meshing with ANSYS-Fluent using a poly-hexcore mesh (a combination of hexahedral elements in the bulk flow region and polyhedral elements), and with eight prism layers to capture the effect of the near wall flow. After a mesh independence study, [Supplementary Data 5], meshes with an element number of 0.3-0.4 Mio elements were created. The maximal cell length was 2e^-3^ m, and the first prism layer thickness was 5.25e^-5^ m. Laminar flow was assumed with a Newtonian fluid and a viscosity of 3 mPas corresponding to a hematocrit value of 23.21±4.1%, which has been measured at the beginning of the experiments. The effect of different hematocrit values and a non-Newtonian blood model on the overall blood flow distribution was found to be negligible in preliminary simulations, justifying the choice of a simplified blood model with a fixed Newtonian viscosity. A fixed volume flow rate (235±12.8ml/min, Figure 2B) was prescribed at the cannula. All vessel boundaries were treated as openings with a "loss coefficient" boundary condition, which allows to consider the resistance of the downstream vasculature. Values were chosen in an iterative process to match flow distribution in the experimental data and set constant for all animals. The individual loss coefficients are listed in [Supplementary Data 5]. Steady state simulations were performed with ANSYS-Fluent using the SIMPLEC as pressure velocity coupling algorithm and a convergence target of 10^-4^.

CFD results were exported, downscaled to the resolution of the PC MRI measurements and postprocessed in Paraview 5.1.2. At first, a comparison between the volume flow rates at the vessel boundaries of the aortic arch and abdominal aorta was performed. In the next step, a centerline starting above the aortic sinus until the renal arteries was created, and the velocity along the centerline was evaluated for CFD and PC MRI. At last, WSS in the aortic arch were compared between antegrade and retrograde perfusion. To allow a comparison with physiological values, the cannula was removed from the CFD models, a generic physiological inflow profile with same mean-flow as the extracorporeal flow was set and the WSS at peak systole was extracted.

**Supplementary Data 5**

**Mesh independence study**

A two-step process was chosen for the mesh independence study. At first, a tetrahedral mesh with prism elements at the walls was created, and the factors element size, total height of the prism layer and the number of the prism layers were varied. The volume flow rate and the area-averaged velocities were selected at the left/right carotid arteries (Lcar/Rcar), left/right subclavian (Lsub/Rsub) arteries and the descending aorta (AoDesc) taken as outputs. The results are shown below in Supplementary Figure 3.

**Supplementary Figure 3:** Velocity and volume flow rate for different mesh settings.

This study yielded a mesh with an element size of 0.2mm, 8 prism layers and a total height of 0.6 mm. In the second step, the mesh was further optimized to reduce the number of elements by employing a poly-hexcore approach which combines hexahedral elements within the domain with polyhedral prism elements at the walls, see Supplementary Figure 4.

**Supplementary Figure 4:** Example of poly-hexcore mesh.

Poly-hexcore meshes use the same element sizes, but have a reduced number of elements, as shown in Supplementary Table 2, which compares different meshing strategies:

**Supplementary Table 2:** Comparison of mesh numbers for tetrahedral and poly-hexcore meshes

|  | **Tetrahedral** | **Poly-Hexcore** |
| --- | --- | --- |
| Boundary layer | 8 | 8 |
| Max. cell length [mm] | 0.2 | 0.2 |
| Number of cells in Mio | 0.84 | 0.33 |

At last, the element size for the poly-hexcore mesh was varied to see whether the results are still mesh-independent. As a control, the volume flow rate at three planes in the aortic arch domains was selected. The results are shown in Supplementary Table 3 and confirm that the max. cell length of 0.2 mm is appropriate.

**Supplementary Table 3:** Mesh settings and volume flow rates at sections A-C

|  | **1** | **2** | **3** | **4** | **5** |
| --- | --- | --- | --- | --- | --- |
| Boundary layer | 8 | 8 | 8 | 12 | 12 |
| First layer height [mm] | 0.0525 | 0.0525 | 0.0525 | 0.0525 | 0.0525 |
| Max. cell length [mm] | 0.2 | 0.1 | 0.05 | 0.1 | 0.05 |
| Number of cells in Mio | 0.33 | 0.4 | 1.17 | 0.48 | 1.5 |
| Flow rate A [ml/s] | 3.08 | 3.08 | 3.09 | 3.08 | 3.09 |
| Flow rate B [ml/s] | 0.279 | 0.279 | 0.273 | 0.279 | 0.273 |
| Flow rate C [ml/s] | 0.242 | 0.242 | 0.238 | 0.242 | 0.238 |

**Loss coefficients**

**Supplementary Table 4:** Loss coefficients for all animals of the aortic arch simulations. Rsub/Lsub: Right/Left subclavian arteries. Rcar/Lcar: Right/Left carotid arteries. DescAo: Descending aorta (only relevant during antegrade perfusion)

| Animal | Rsub | Rcar | Lsub | Lcar | DescAo |
| --- | --- | --- | --- | --- | --- |
| Antegrade perfusion (n=4) | | | | | |
| 0475 | 500 | 150 | 65 | 9 | 25 |
| 0652 | 650 | 3 | 8 | 180 | 8 |
| 0653 | 14 | 0.5 | 180 | 60 | 30 |
| 0654 | 100 | 140 | 20 | 80 | 13 |
| Retrograde perfusion (n=4) | | | | | |
| 0476 | 110 | 130 | 7 | 20 | - |
| 0477 | 35 | 6 | 50 | 10 | - |
| 0478 | 69 | 230 | 100 | 16 | - |
| 0588 | 60 | 20 | 300 | 1 | - |

**Supplementary Table 5:** Loss coefficients for all animals of the abdominal aorta simulations. TrCoe: Coeliac trunk. Sma: superior mesenteric artery. Rra/Lra: Right/Left renal arteries. AoAbd: Abdominal aorta

| Animal | TrCoe | Sma | Rra | Lla | AoAbd |
| --- | --- | --- | --- | --- | --- |
| Antegrade perfusion (n=3) | | | | | |
| 0475 | 2 | 8 | 50 | 2500 | 40 |
| 0653 | 18 | 35 | 22 | 30 | 56 |
| 0654 | 5.1 | 5 | 10 | 98 | 39 |
| Retrograde perfusion (n=3) | | | | | |
| 0477 | 1 | 20 | 20 | 55 | 18 |
| 0478 | 4 | 21 | 15 | 20 | 35 |
| 0588 | 200 | 200 | 5 | 60 | 75 |
