# Supplementary material for: Validation of Aortic Blood Flow Simulations During Extracorporeal Circulation Using Phase Contrast Magnetic Resonance Imaging
Source: Artif Organs. 2026 Apr 23;50(9):1217–27. doi: 10.1111/aor.70136 (PMC13525229; doi:10.1111/aor.70136)
Supplement: Supplementary file 2 [file AOR-50-1217-s003.docx]

**Supplementary Figures**

**Supplementary Figure 1**

**Hemodynamic parameters and oxygenation under ECC**

**A**: Hemoglobin levels (median+IQR) before and during extracorporeal circulation (ECC). **B**: Hematocrit levels (median+IQR) before and during ECC. **C**: Blood flow rates (median+IQR) during ECC. **D**: Mean arterial pressure (MAP) (median+IQR) before and during ECC. **E**: Arterial partial pressure of oxygen (paO_2_) (median+IQR) before and during ECC. **F**: Peripheral oxygen saturation (SpO2) (median+IQR) before and during ECC.

**Supplementary Figure 2**

**Inflammatory activity and brain damage markers**

**A**: Comparison of neuronspecific enolase (NSE) in rabbits with physiological blood flow, antegrade or retrograde perfusion before and after the MRI measurement. **B**: Comparison of S100 calcium-binding protein (S100) in rabbits with physiological blood flow, antegrade or retrograde perfusion before and after the MRI measurement. **C**: Interleukin-6 gene expression of controls, of rabbits with physiological blood flow, antegrade or retrograde perfusion. **D**: Tumor necrosis factor alpha (TNF alpha) gene expression of controls, of rabbits with physiological blood flow, antegrade or retrograde perfusion. **E**: Ionized calcium-binding adapter molecule 1 (IBA1) gene expression of controls, of rabbits with physiological blood flow, antegrade or retrograde perfusion. **F**: NSE gene expression of controls, of rabbits with physiological blood flow, antegrade or retrograde perfusion. **G**: S100b gene expression of controls, of rabbits with physiological blood flow, antegrade or retrograde perfusion.
