# Supplementary material for: Validation of Aortic Blood Flow Simulations During Extracorporeal Circulation Using Phase Contrast Magnetic Resonance Imaging
Source: Artif Organs. 2026 Apr 23;50(9):1217–27. doi: 10.1111/aor.70136 (PMC13525229; doi:10.1111/aor.70136)
Supplement: Supplementary file 3 [file AOR-50-1217-s004.docx]

**Supplementary Table**

**Supplementary Table 1**

| **Primer** | **Forward Sequence** | **Reverse Sequence** |
| --- | --- | --- |
| β_2_M | 5'- ATGAGTATTCCTGCCGGGTG -3' | 5'- GGTATCCTCAGACCTCCATGC -3' |
| IBA1 | 5'- AACCTTGAGCGTGGGGATTG -3' | 5'- GCCGTTGTAGTGAAGTGAGGA -3' |
| IL-6 | 5'- ACGGTCAGAACACACCATCC -3' | 5'- GTGTCCTAACGCTCATCTTCCT-3' |
| NSE | 5'- GATGGGACTGAGAACAAATCCAAG -3' | 5'- TGGCGATACAGGGGCAACTC -3' |
| S100B | 5'- GAAGGGAGGGAGACAAGCAC -3' | 5'- CCTCCTGCTCTTTGATTTCCTCT -3' |
| TNF alpha | 5'- ACATCACCGAACCTCTGCTC -3' | 5'- AGGAGTCTTTATTTCTCGCCAC-3' |

**Primer sequences for quantitative RT-PCR**

β2M, beta-2 microglobulin; IBA 1, ionized calcium-binding adapter molecule 1; IL-6, interleukin-6; NSE, neuronspecific enolase; S100B, S100 calcium-binding protein B; TNF alpha, tumor necrosis factor alpha.
